# Bioprinting of kidney *in vitro* models: cells, biomaterials, and manufacturing techniques

**DOI:** 10.1042/EBC20200158

**Published:** 2021-08-06

**Authors:** Maaike F.J. Fransen, Gabriele Addario, Carlijn V.C. Bouten, Franck Halary, Lorenzo Moroni, Carlos Mota

**Affiliations:** 1 MERLN Institute for Technology-inspired Regenerative Medicine, Department of Complex Tissue Regeneration, Maastricht University, the Netherlands; 2 Department of Biomedical Engineering, Institute for Complex Molecular Systems (ICMS), Eindhoven University of Technology, the Netherlands; 3 Nantes Université, Inserm, CHU Nantes, Center for Research in Transplantation and Immunology, 11 UMR1064, ITUN, Nantes, France

**Keywords:** biomaterials, Bioprinting, in vitro models, kidney

## Abstract

The number of patients with end-stage renal disease is continuously increasing worldwide. The only therapies for these patients are dialysis and organ transplantation, but the latter is limited due to the insufficient number of donor kidneys available. Research in kidney disease and alternative therapies are therefore of outmost importance. *In vitro* models that mimic human kidney functions are essential to provide better insights in disease and ultimately novel therapies. Bioprinting techniques have been increasingly used to create models with some degree of function, but their true potential is yet to be achieved. Bioprinted renal tissues and kidney-like constructs presents challenges, for example, choosing suitable renal cells and biomaterials for the formulation of bioinks. In addition, the fabrication of complex renal biological structures is still a major bottleneck. Advances in pluripotent stem cell-derived renal progenitors has contributed to *in vivo*-like rudiment structures with multiple renal cells, and these started to make a great impact on the achieved models. Natural- or synthetic-based biomaterial inks, such as kidney-derived extracellular matrix and gelatin-fibrin hydrogels, which show the potential to partially replicate *in vivo*-like microenvironments, have been largely investigated for bioprinting. As the field progresses, technological, biological and biomaterial developments will be required to yield fully functional *in vitro* tissues that can contribute to a better understanding of renal disease, to improve predictability *in vitro* of novel therapeutics, and to facilitate the development of alternative regenerative or replacement treatments. In this review, we resume the main advances on kidney *in vitro* models reported so far.

## Introduction

Chronic kidney disease (CKD) is the long-term loss of kidney function, which, in 2017, affected globally 697.5 million people [1,2]. The common endpoint of CKD is renal fibrosis, which is characterized by an increase in extracellular matrix (ECM) deposition [2]. With the progression of CKD, patients that reach an ultimate disease stage termed end-stage renal disease are placed on life-saving dialysis therapy while waiting for a suitable donor kidney [3–5]. However, kidney transplantation is limited by the insufficient number of available healthy donor kidneys.

To account for kidney shortage, much effort has been put to manufacturing bioengineered kidneys or units that could improve renal function by replacing or complementing a kidney, respectively [3]. Although the achievement of these units is still far from reality, researchers have focused on developing *in vitro* models as platforms to investigate renal disease and alternative regenerative or replacement therapies. Renal *in vitro* models have been manufactured using bioprinting technology, which allows to build three-dimensional (3D) constructs combining cells, biomaterials, and other biological factors in pre-designed strategies. However, bioprinting renal constructs presents various challenges: (i) the choice of renal cell type(s), (ii) the choice of biomaterial(s), and (iii) the difficulty to produce the complex renal architecture that preserves the biological structure while facilitating tissue maturation and function post-printing [3]. *In vitro* models are essential to investigate renal diseases and the discovery of new therapies. These *in vitro* models will give insights into the future possibility to build functional organs, which scientists strive for.

Bioprinted renal models achieved so far will be reviewed, with focus on manufacturing techniques, kidney cells, and biomaterials. Furthermore, other models such as organoids and organ-on-chip will be briefly covered as these have been combined in a synergistic way to allow further maturation, tissue mimicry, and function. Finally, an outlook is provided where multiple points of consideration are highlighted for future developments.

## Bioprinting techniques

**Figure 1:**
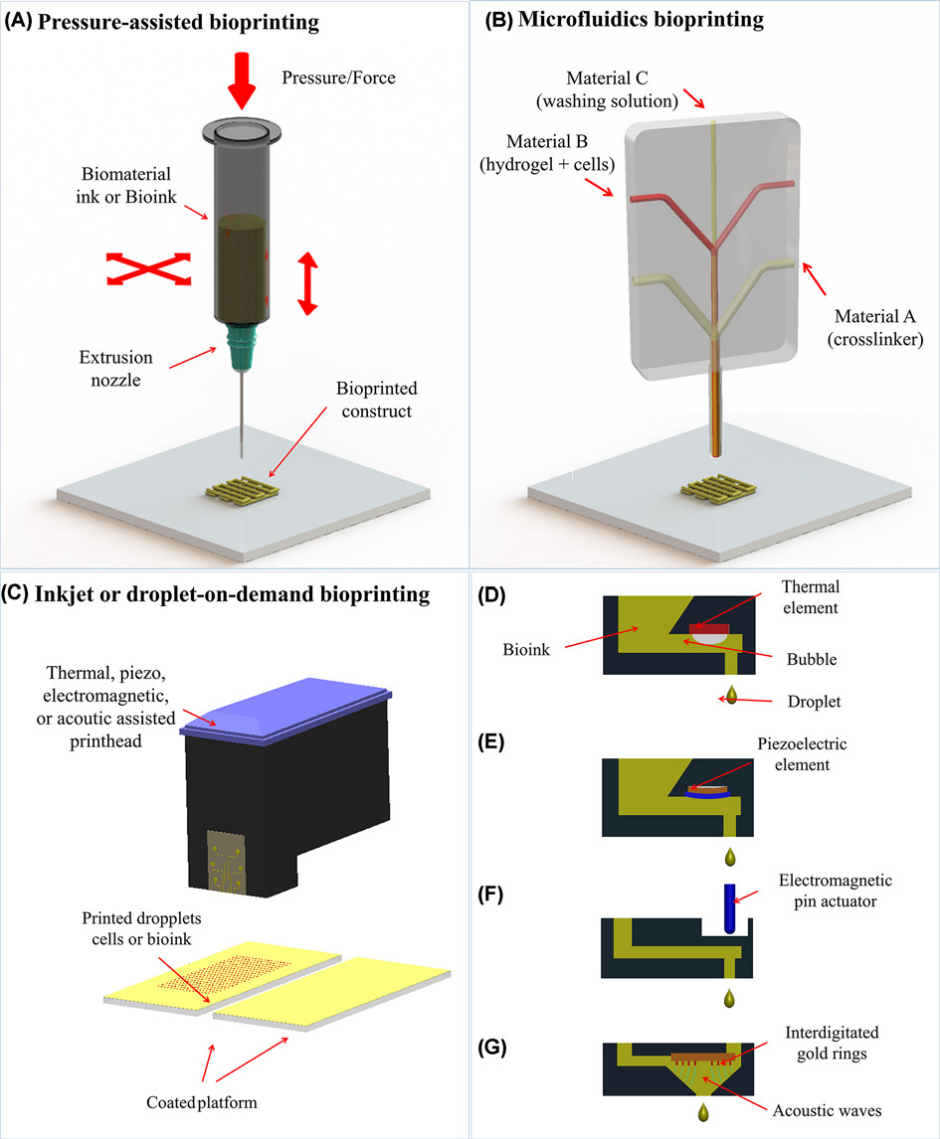
Bioprinting techniques used to create kidney *in vitro* models (**A**) extrusion-based (pressure-assisted), (**B**) microfluidics, and (**C**) inkjet or droplet on demand bioprinting with (**D**) thermal, (**E**) piezo, (**F**) electromagnetic, or (**G**) acoustic actuation. Adapted with permission from Mota et al. [6].

Bioprinting is a group of additive manufacturing (AM) technologies that allows the precise deposition of biomaterial inks or bioinks (see biomaterial inks or bioinks section) layer-by-layer to fabricate complex tissues or organ models [6–8]. To achieve relevant kidney models, the cells, biomaterial and/or bioactive molecules should be carefully selected [9]. The 3D model is manufactured using bioprinting techniques and matured in a bioreactor or directly implanted *in vivo* [9,10]. Various bioprinting techniques have been developed in the past decades and mainly two groups, namely extrusion- or microfluidics-based and droplet-on-demand have been used to create kidney models (Figure 1).

Extrusion-based bioprinting are techniques that produce a single stream of material and are commonly used to dispense filaments of fugitive biomaterial inks or bioinks [8,9,11]. The ink is extruded through a nozzle by air or gas pressure (Figure 1A) or by mechanical displacement, i.e., with a piston or a screw [9]. These techniques are relatively simple and cheap, allowing to print high cell density and higher viscosity solutions [9]. However, higher viscous bioinks (above 30 mPa·s) can cause shear stress-damage to the cells during bioprinting, resulting in low cell viability [9,11–13].

Taking into account the main drawbacks of extrusion-based bioprinting, new systems such as microfluidic bioprinting started to be developed that allow the co-extrusion in a core–shell format (Figure 1B). Bioinks are simultaneously dispensed with crosslinking solution allowing a drastic reduction of shear–stress as the formed bioink filament is never in direct contact with the nozzle surface [14–16]. Furthermore, polymeric solutions with low concentrations and viscosities are normally used, allowing to produce filaments with diameters ranging from 50 µm to 1 mm with high cell viability. Extrusion-based bioprinting (pneumatic driven and microfluidics) is generally frequently used for the manufacturing of kidney *in vitro* models.

Inkjet-based or droplet-on-demand (DOD) bioprinting, dispenses droplets of biomaterials ink or bioink from a cartridge onto a substrate (Figure 1C) [6,17]. The droplet formation can be triggered by temperature, piezoelectric element deformation, electromagnetic actuation, or acoustic waves (Figure 1D–G). Once formed, the droplet is projected against a moving platform being the construct produced from an accumulation of droplets selectively dispensed. Inkjet-based bioprinting ensures a high resolution (∼50 µm) while dispensing single droplets containing cells in a microarray or scaffold formats [18]. DOD technology has been applied to renal cell lines that were able to proliferate after the bioprinting process [19]. In the present study, bioprinted droplets were selectively dispensed inside an oil bath that once removed was replaced with a stabilization hydrogel to maintain the construct integrity while tissue maturation occurred.

## Kidney cells

**Figure 2:**
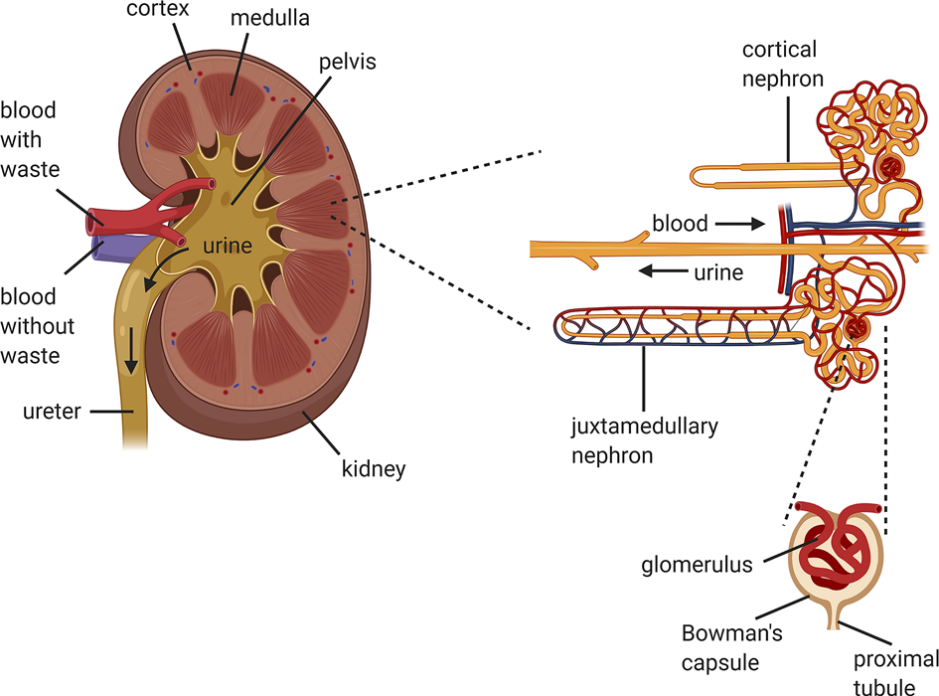
Anatomy of the kidney Schematic representation of the anatomy of the kidney with details of the nephrons and glomerulus compartment where the filtration occurs. Created with Biorender.com.

The kidney is a complex organ that comprises around 1 million of functional units termed nephrons that continuously filter the blood, eliminating metabolic waste products, while maintaining homeostasis of body fluids by producing hormones (Figure 2) [6,7,20]. Each nephron consists of more than 20 different cell types, e.g., glomerular podocytes, proximal and distal tubule epithelial cells, collecting duct cells, and many others [7,21]. In kidney models produced by bioprinting, mainly two different cell populations are used, epithelial [20,22–25] and endothelial cells [20,24,26], to mimic the proximal renal tubule and the intricate exchange with the vasculature in its vicinity. Cell lines are mainly used due to their availability, established culture protocols, and reduced costs. Primary cells have also been used as they preserve the normal morphology and important markers and functions but present limitation in terms of expansion and pure populations are still challenging to obtain. Despite the utilization of human primary cells would be the closest approach to develop relevant models, these cells can only be isolated from organs. This limits their origin from organs rejected for transplantation or from resected organs that are normally at a late disease stage where both cellular and acellular composition is largely affected [6]. Generally, cell lines [19,20,27,28] have been used to manufacture the earliest models, while in some more recent examples primary cells isolated from animal [14] or human kidneys [24,25,29] have been used. Alternatives such as human pluripotent stem cells (PSCs) have been used to generate renal progenitor and organoids.

In the kidney, epithelial cells play an active role in reabsorption of water, ions and other small molecules, and in the concentration of the filtrate. Most studies reported in literature use proximal tubule epithelial cells (PTECs) as the proximal tubule (PT) is the primary site damaged by xenobiotics, which can cause irreversible reduction of kidney function [23,24]. In most studies, unmodified PTECs were used for a PT *in vitro* model [20,24,30]. In other studies PTEC-TERT1 cells [23,25], human kidney PT epithelial cells (HK-2) [20] and HEK293 [19,28] were used since they are robust and fast-growing cells. Other examples of used epithelial cells are animal-derived immortalized cells such as MDCK-1 [27,31].

Endothelial cells form the internal lumen of the vascular system and have an essential function in the blood supply to surrounding tissue. Within the kidney, different endothelial regions have distinctive functions. For instance, the intricate network of the glomerular endothelium is where a primary filtrate occurs within the Bowman’s capsule, while microvascular endothelium forms the peritubular capillaries that extend throughout the nephron and its primary function is the reabsorption of water and other solutes recovered by the nephrons epithelium [6,32]. Diverse studies used human umbilical vein endothelial cells (HUVECs) to fabricate a more complex renal model, i.e., to mimic the PT interstitial interface by including vascular tubules [20,24,26]. Glomerulus microvascular endothelial cells (GMECs) have also been investigated to form tubular structures instead of HUVECs [25]. Furthermore, endothelial cells from rat primary veins have been used to produce an *in vitro* model to study the glomerular filtration barrier in combination with podocytes [33].

Podocytes are a specialized cell population present in the glomerulus that cover the glomerulus capillary network. Their main function is to prevent plasma proteins to enter in the filtrate [34]. Recently, protocols for the differentiation of induced pluripotent stem cells (iPSCs) toward podocytes have also been established [35–37]. These iPSCs-derived podocytes were used on a dual chamber on-chip mimicking the glomerulus, which showed improved barrier function when cultured with primary glomerular endothelial cells on each side of a polydimethylsiloxane (PDMS) membrane [36]. Other examples used are murine podocyte immortalized cell line (E11, Biocat) that improve the barrier function on a fluidic perfusable model by involving a tubular lumen of endothelial cells [33]. Although the application of podocytes in bioprinted models has not been reported so far, they might be essential to be able to achieve improved function.

Fibroblasts are cells dispersed throughout the body and thereby they are the least specialized connective tissue cells although recent studies demonstrated an unprecedented complexity/specialization of the adult skin fibroblasts [38,39]. Fibroblasts secrete diverse ECM structural and adhesive proteins, and ground substances to ensure a balance of ECM composition. In the kidney, fibroblasts have an endocrine role and thereby secrete erythropoietin, which is a critical hormone in the maintenance and formation of red blood cells [40–42]. Furthermore, fibroblasts have an active role during kidney injury exacerbating inflammation and fibrosis [43]. Reports of the inclusion of fibroblasts in the *in vitro* models are limited. King et al. combined human renal fibroblasts, with PTECs and HUVECs, demonstrating that fibroblasts contributed to the newly deposited ECM and supported the formation of an open channel vascular network [24].

Primary cell populations are most likely the best choice to achieve a better degree of function *in vitro*, since multiple cells type are collected during isolation [44]. The need for a donor organ limits their application for tissue engineering and regenerative approaches, as well as for *in vitro* models. Ali et al. used isolated human primary kidney cells combined with decellularized metacrylated ECM to form bioinks [29]. The cells were able to reorganize inside the bioprinted constructs after 2 weeks in culture exhibiting tubular and glomerular-like structures with a certain degree of reabsorption and other barrier and transport functions [33,45].

Stem cells (SCs) are unspecialized cells, which are able to differentiate into other cell types and have the ability to self-renew [46]. Pluripotent stem cells (PSCs) can differentiate into cells of all germ layers, e.g., embryonic stem cells (ESCs), and thereby have great potential in organ regeneration and bioengineering [45]. As the research with ESCs raises several ethical concerns, alternative cells such as iPSCs, i.e., adult cells reprogrammed to an ESC-like state, generated by the dedifferentiation of adult cells from primary tissues are a promising alternative. In 2012, a protocol was established for the differentiation of iPSCs into renal podocyte-like cells and this initiated the establishment of various protocols for the differentiation of PSCs toward different renal-like cell types and renal lineage precursor-like cells [35]. Multiple reported protocols are nowadays available for the differentiation of embryonic and induced PSCs toward renal progenitor and kidney organoids [47,48]. Kidney organoids are self-organizing 3D kidney rudiment structures that show some degree of anatomical and functional hallmarks of a kidney at early development stages [3,11,47]. Human PSC-derived kidney organoids yield the potential to be used in kidney transplantation although the early development stage and the limitations in maturation are still limiting their translation [49–51]. Further optimization is necessary before organoids can be used in the clinic as these contain up to 20% of nonrenal cell types [52]. Initial studies have investigated the use of PSCs and more specifically iPSC-derived organoids with a bioprinting method [50]. This initial approach aimed at producing a screening platform in a 96 well plate format for drug-induced nephrotoxicity [50]. Although the reports on the combination of organoids and bioprinting are still limited further progress should be expected in the coming years.

## Biomaterial inks and bioinks

In biofabrication, and more specifically bioprinting, a distinctive definition for biomaterial inks and bioinks has been established [53]. A bioink is a biomaterial, in most cases a hydrogel, which contains cells. Conversely, the biomaterial ink does not include cells within the process and these are generally seeded post-bioprinting. In renal bioprinting, the biomaterials used to form bioinks serve as cell microenvironment during and after the bioprinting of a construct, while facilitating a certain degree of maturation and function post-bioprinting.

The requirements of a biomaterial ink and bioink depend on the bioprinting technique. The biomaterials used can be classified in the following groups: polymers (thermoplastics, elastomers and hydrogels) and composites [6]. Thermoplastic polymers, elastomers and some composite formulations cannot be used for bioink formulations and are generally used to manufacture 3D supporting structures for softer hydrogels materials [45] or as a bioreactor chamber [23]. These groups of materials generally require processing conditions that are not compatible with cells (e.g., high processing temperature and pressure).

Thermoplastic polymers become ductile above the melting temperature and return to a solid state below the crystallization temperature [54]. In bioprinting renal *in vitro* models the thermoplastic polymer mainly used is poly(ε-caprolactone) (PCL) due to its relatively low melting temperature (∼60°C). This polymer is normally processed with fused deposition modeling (FDM) and used as a scaffold structure for a bioink [55] or to create chips for perfusion of a bioprinted kidney *in vitro* model [20].

Elastomers are only used in bioprinting to create printed chips for perfusion as these materials are generally not biocompatible nor biodegradable. Dual component silicone elastomer has been used to produce a container to confine a casted hydrogel around pluronic fibers used to create perfusable channels [22,23]. These dual component silicone elastomers are normally inert and nontoxic when completely crosslinked and have been extensively used in the microfluidics field [56].

Hydrogels are networks of hydrophilic polymer chains dissolved in aqueous solutions and have multiple advantages such as biocompatibility, tunable biodegradability, and highly porous structure [57,58]. The optimal hydrogel formulation requires a fine balance between the aimed bioprinting resolution and optimal cellular behavior post-bioprinting which limits the range of processing conditions to satisfy both needs [59]. Hydrogels can be classified on the basis of natural or synthetic. While natural-based hydrogels have the advantage to mimic the biological microenvironment of the native ECM, the synthetic counterparts allow a broader range of modifications, such as chemical functionalization and overall control over the physicochemical properties [29,58,60].

One of the most investigated natural-based material used for bioprinting is decellularized ECM (dECM) as it can be used to form hydrogel with tissue-specific composition and biophysical properties that support cell proliferation, adhesion, differentiation, and maturation [20,29]. The main disadvantages of the use of dECM is the need of a donor organ or tissue and the multiple steps necessary for the decellularization and partial digestion for being able to form a bioprintable solution and subsequently a stable gel. Furthermore, dECM lacks tunable biochemical composition, has very low viscosity, and the crosslinking strategies and compatibility as stand-alone material for bioprinting is still limited. Chemically modification of the dECM by methacrylation has been proposed as a suitable photo-crosslinkable strategy to produce solutions that can be bioprintable [29]. Other natural-based materials from animal or plant source used to form hydrogels for bioprinting on renal *in vitro* models are gelatin [14,22,23,25,28,29], agarose [19], hyaluronic acid [29], glycerol [29], sodium alginate [14,20,28], and fibrin [22–24,28]. Another natural-based hydrogel largely used when PSC-derived organoids or renal progenitors are employed is sarcoma cell-secreted protein ECM (e.g., Matrigel and Geltrex) [61,62]. Similarly to organ or tissue-derived ECM these cell-derived ECM hydrogels are not suitable for most bioprinting techniques and are normally used as substrate to expand renal progenitors or to form organoids [48,50,63].

Synthetic hydrogels generally used for the manufacturing of renal *in vitro* models are mainly used as sacrificial materials. Pluronic is frequently a material of choice as this can be processed with extrusion bioprinting to produce sacrificial filaments to form empty channels or networks of channels inside a casted hydrogel [23,25,64]. Another example of pluronic application is in core–shell bioprinting strategies where bioinks are extruded concentrically with a pluronic solution in the core of a filament. Once the structure is crosslinked, a hollow fiber can be achieved by removing this sacrificial material [20]. The removal is done by inducing a phase transition from a gel to liquid by cooling the bioprinted constructs at 4°C [22,23].

## Kidney *in vitro* models

Two-dimensional (2D) cell cultures are largely used for nephrotoxicity screening during drug development but these do not accurately recapitulate the 3D microenvironment and function of adult human kidney [65,66]. Therefore, 3D models capable of mimicking closely kidney functions in a more physiologically relevant way are continuously studied, not only envisioning the improvement in organogenesis and organoid maturation for novel therapies but also to improve drug discovery and nephrotoxicity screening or to study disease *in vitro* [20,24,25,50,51,67,68]. While bioprinted kidney *in vitro* models are being proposed, similarly important models such as kidney-on-chip and organoids are also briefly covered. The principles applied to these somewhat overlap and will likely contribute for *in vitro* models that are more mature, physiological, and functionally relevant in the near future.

**Figure 3:**
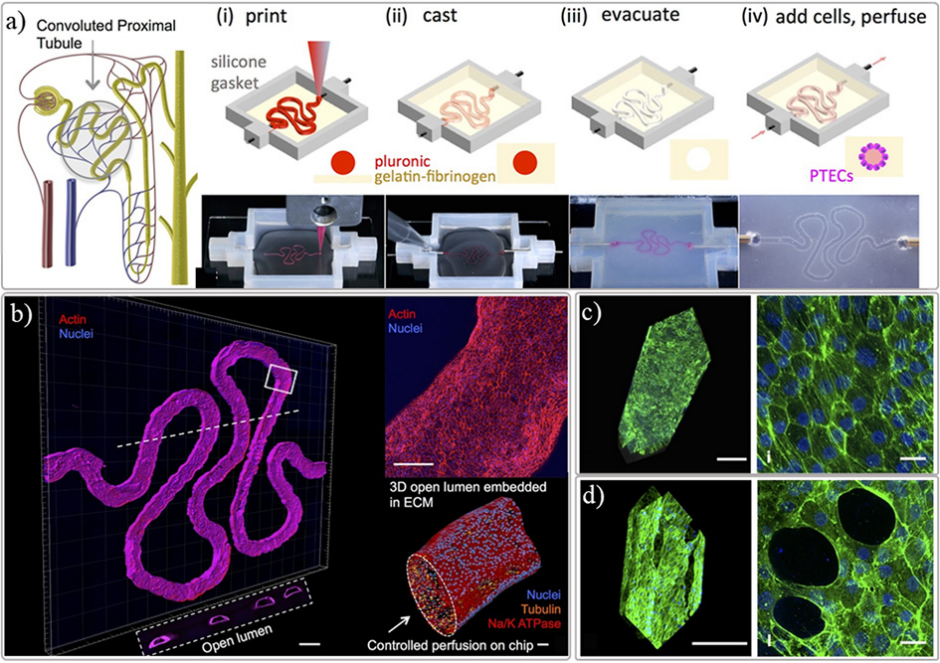
Renal proximal tubule *in vitro* model within perfusable chip ( **A**) manufacturing strategy using sacrificial 3D printing to create hollow channel after post-encapsulation with a gelatin-fibrin extracellular matrix hydrogel, (**B**) proximal tubule-like fully epithelialized after post-seeding and cultured with proximal tubule epithelial cells (PTECs), (**C**) control group of tubule model used to screen the toxicity effect of cyclosporine A and (**D**) disrupted epithelial barrier observed when model was exposed with a high concentration (100 μM) of the chemical compound. Adapted with permission from ref. [23].

**Figure 4:**
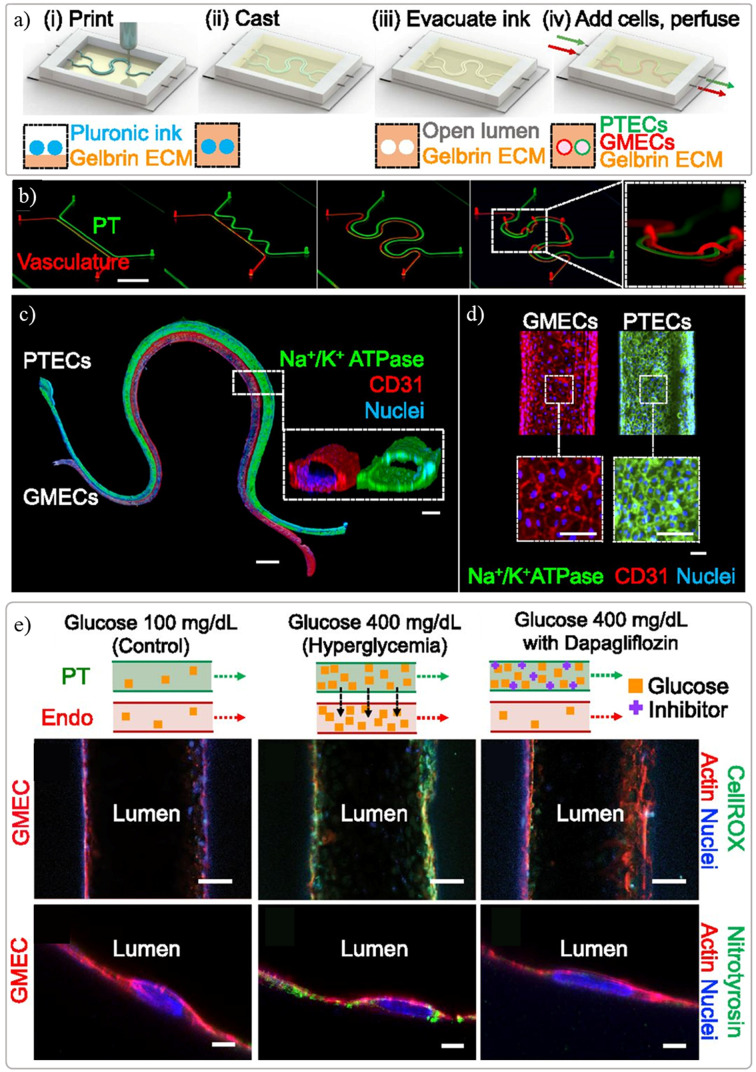
Renal proximal tubule model with parallel vascular channel ( **A**) manufacturing strategy using sacrificial 3D printing to create two parallel hollow channels after post encapsulation with a gelatin-fibrin extracellular matrix hydrogel, (**B**) tubules can be manufactured in different configurations, including out of the plane, (**C**) full endothelialized and epithelialized tubules stained with cell-specific markers obtained after post-seeding with respective cells and cultured under perfusion up to 5 days, and (**E**) functionality test performed on the model to evaluate the influence of hyperglycaemic conditions on filtration and reabsorption response with and without the presence of a glucose transport inhibitor. Adapted with permission from ref. [25].

**Figure 5:**
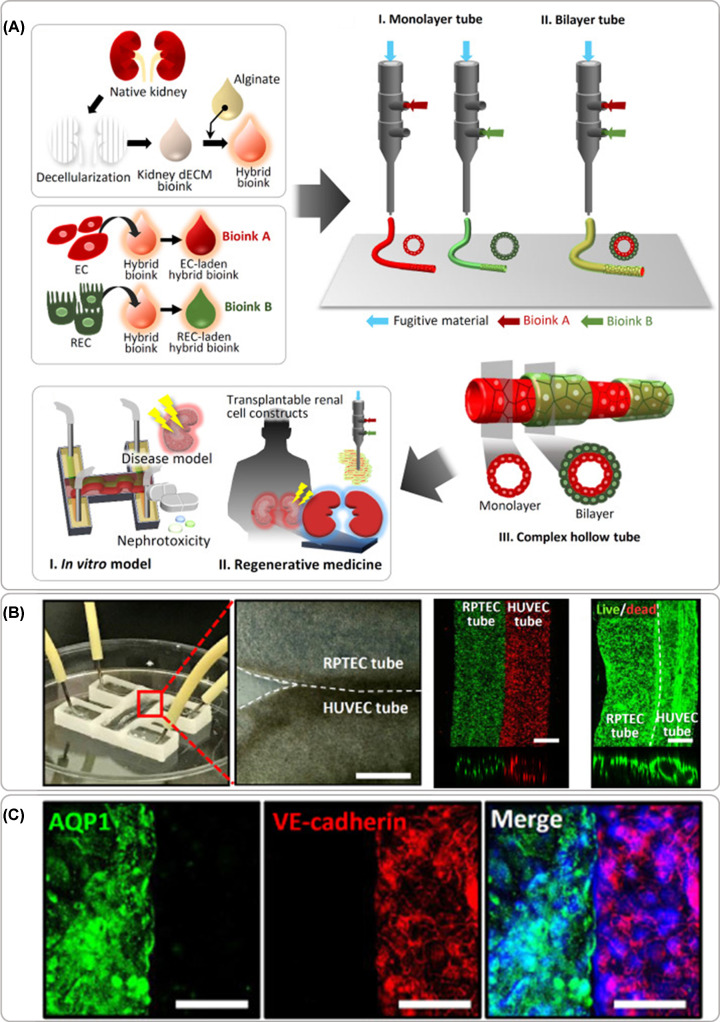
Bioprinted renal tubule model ( **A**) Schematics of the manufacturing approach combining kidney decellularized extracellular matrix with and alginate with endothelial or epithelial cells to form bioinks dispensed with core–shell bioprinting approach with individual cells to form tubular constructs or multi-shell arrangements, and the envisioned applications, (**B**) perfusable model manufactured with parallel epithelial and endothelial tubules in close contact showing high cellular viability post printing and hollow channels with a cellular lumen, and (**C**) immunostaining of cell specific markers. The barrier function of cell layers was tested *in vitro* and implanted *in vivo* under the kidney capsule envisioning future regenerative medicine application. Adapted with permission from ref. [20].

Despite the ambition of using tissue assembly and bioprinting to manufacture kidney tissue dates back to 2006 [69], little progress has been made until recently where an exponential increase of research has been observed. This slow progress was associated with the challenges also identified by Mironov et al., where biological, vascularization, and technological aspects were not sufficiently developed to achieve a functional bioprinted kidney tissue [69]. The first noteworthy steps toward a kidney *in vitro* model were published in 2016 [23,64], where the Lewis lab used sacrificial bioprinting with fugitive material to produce networks of centimeter-sized vascularized constructs [64], but also a fully epithelialized channel mimicking the proximal tubules (Figure 3) [23]. The same group reported a dual channel perfusable kidney model following the same sacrificial bioprinting principles to produced endothelialized and epithelialized channels where some degree of function (filtration and reabsorption) was observed between the two parallel channels (Figure 4) [25]. In these models, a mixture of gelatin and fibrin was used as ECM material to cast over the bioprinted sacrificial filament, which was subsequently liquefied and removed leaving an empty channel. A postseeding step allows the creation of the lumen of cells without the need to include cells in the bioprinting process [22,23,25,64]. Singh et al. reported a similar *in vitro* models with two manufacturing approaches using renal PTECs and HUVECs to create tubules with a core-shell bioprinting approach where an external shell included cells or bioinks and the core contained pluronic F127 (Figure 5) [20]. The first approach involved the concentric bioprinting of a shell with endothelial cells wrap around with a second shell with epithelial cells and a core made of pluronic to mimic the glomerulus/proximal tubule sections. A second approach followed a similar model, as previously described, with two parallel perfusable channels. The inlets and outlets were fixed with PDMS, culture media was added to the chip, and the fugitive ink was liquefied and perfusion was done with culture media [20]. A good tubular structures with a uniform cellular lumen where the filtration and reabsorptions mechanisms known to a nephron unit were tested *in vitro*.

**Figure 6:**
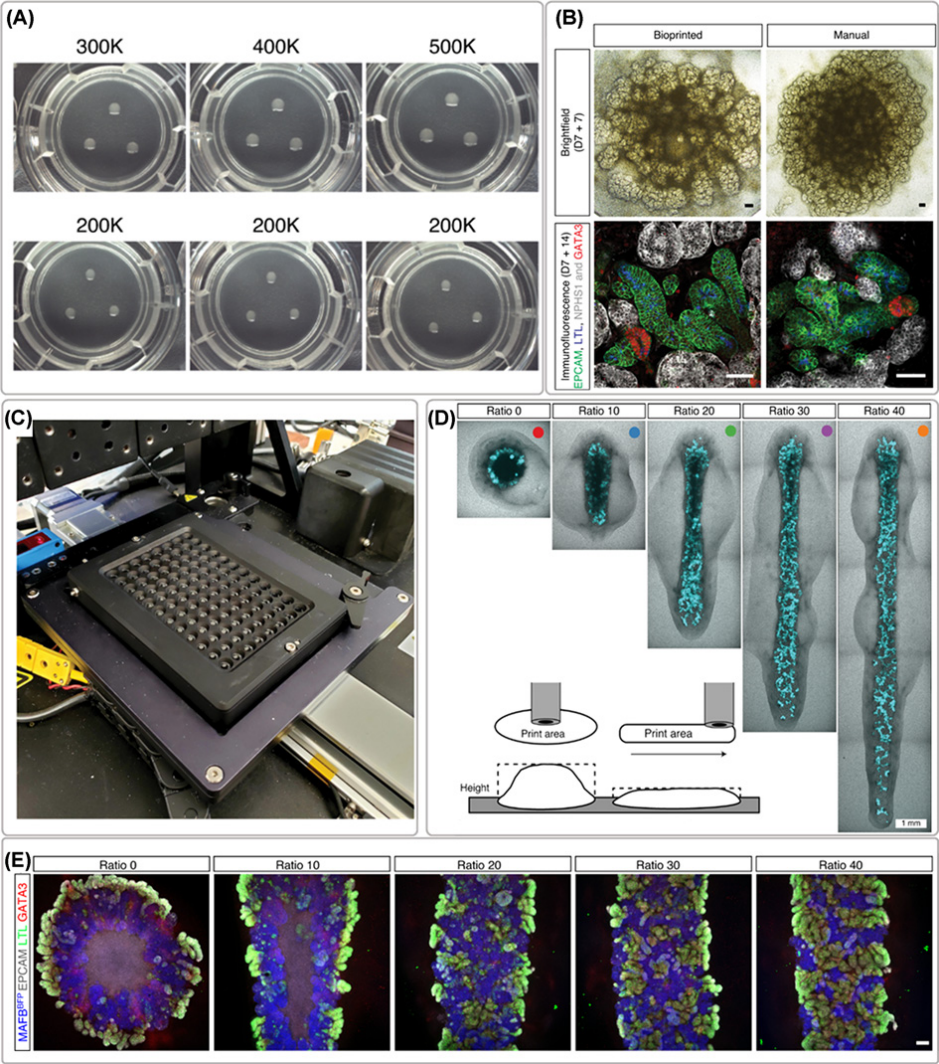
Bioprinting of iPSCs-derived renal cells for the formation of organoids ( **A**) example of organoids formed after printing different cell numbers (300,000–500,000 cells) and the reproducibility test with 200,000 cells, (**B**) brightfield and whole-mount immunofluorescence morphological comparison between bioprinted and manually formed organoids, (**C**) 96-well plate platform for compound testing, (**D**) organoid conformation studies, and (**E**) immunofluorescence staining of organoids created with different length while maintaining the same number of cells showing thinner cell mass with improved morphology. Adapted with permission from ref. [50].

As mentioned before, the biological limitations were also investigated and more relevant and complex cellular models have become available. Kidney organoids or self-organizing 3D kidney rudiment structures are one of the most advanced examples used to develop *in vitro* models for disease modeling and drug screening studies [11]. High cell density paste have been dispensed with extrusion bioprinting to form kidney organoids in a 96 well plate platform for high throughput drug-induced nephrotoxicity testing (Figure 6) [50]. This platform containing organoids with a higher reproducibility in terms of size, conformation and cells number resulting in the improvement of nephron maturation. The use of bioprinting allowed a 15–20 times faster production than manual techniques for the same amount of organoids [50].

**Figure 7:**
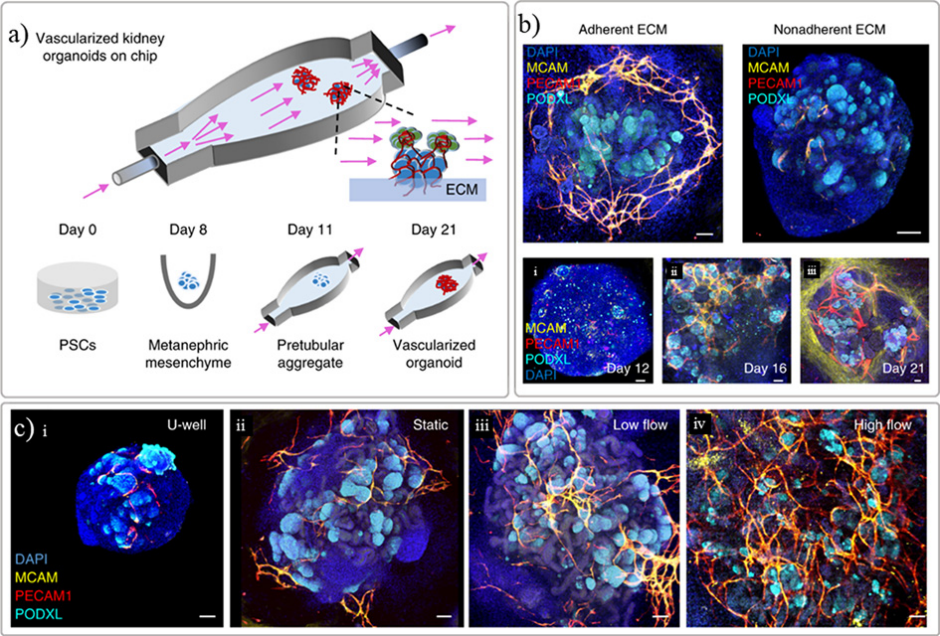
Maturation of renal organoids *in vitro* with a perfusable chip (**A**) schematic representation of the manufactured chip where PSC-derived kidney organoids are seeded onto a prevascularized ECM bed, (**B**) results demonstrating that the selection of ECM is important for the success of the vascularization, and (**C**) the importance of fluid-flow on the vascularization and growth of the organoids as demonstrated by endothelial and podocyte markers (MCAM, PECAM1, and PODXL). Adapted with permission from ref. [70].

Generating bioprinted functional kidneys patches for transplantation will be a long-term goal as organoid models still lack suitable maturation levels [49,50]. This limitation can be circumvented by implementing flow-enhanced vascularization and environmental cues [49,63,70]. The maturation of the kidney organoids could also be improved upon transplantation *in vivo* as the host-derived endothelial cells can initiate vasculogenesis within the transplant [71]. Some of the *in vitro* examples previously reported include principle of organ-on-chip platforms to improve perfusion, maturation, and function. Kidney-on-chip is the combination of a kidney cell culture within a platform, i.e., microfluidic chip, recreating a more realistic, controlled microenvironment *in vitro* to study renal disease, and drug screenings [4,8,11,72]. Organ-on-a-chip are generally fabricated using soft lithography techniques to make PDMS chips in which cells can be seeded [11]. This method allows the production of diverse chip designs, which can mimic *in vivo* renal physiology. Diverse renal tissue chips have already been manufactured, such as glomerulus-on-chip and tubule-on-chip [3,73,74]. For example, a glomerulus-on-chip mimicking the glomerular human filtration barrier was studied *in vitro* using podocytes and glomerular endothelial cells seeded on multichannel chip Organoplates™ [73]. Perfusable chips were also produced by printing an elastomer (Figure 7A) that were used to enhance the maturation of organoids, where the selection of ECM (Figure 7B) and fluid-flow (Figure 7C) played a key role on vascularization, as proved by endothelial and podocytes markers [70]. Furthermore, platforms that allow a higher throughput or number of conditions such as commercial or customized multiwell-like plates, offer advantages like having compatibility with multiple laboratory equipment while allowing to screen a significant amount of conditions at a time [33,50,73,75]. These platforms have been combined with bioprinting or automated liquid dispensing units to allow higher throughout while offering a finer control on the biologicals dispensed in each well. These higher throughput platforms facilitate the screening of kidney organoids growth conditions envisioning further maturation stages but also the possibility to screen libraries of compounds in a faster and easier way. Examples of the kidney *in vitro* models reported so far using different bioprinting techniques envisioning multiple applications are resumed in Table 1.

**Table 1:** Resume table with comparison of the bioprinted renal models developed so far

Renal compartment	Cells	Biomaterial	Bioprinting technique	Model description	Advantages	Disadvantages	Ref.
Proximal tubule model	HUVECs, PTECs, HK-2 cells	dECM, sodium alginate, pluronic	Extrusion-based	Bioprinted monolayer and bilayer cell tubules	Cells bioprinted in different concentric configurations; Function demonstrated	Resolution above 500 µm; dECM requires source organ	[20]
Proximal tubule model	HUVECs, renal fibroblast, PTECs	Thermo-responsive proprietary biomaterial ink	Extrusion-based (piston-driven)	Bioprinted cell-laden bioink onto a transwell membrane	Barrier function demonstrated; Suitable for nephrotoxicity studies	Layered model not compatible with perfusion	[24]
Proximal tubule model with and without vasculature	PTECs, GMECs, HUVECs	Gelatin, fibrin, pluronic	Extrusion-based	Printing of fugitive ink filaments Encapsulation with hydrogel Seeding hollow channels after removal of filaments	Demonstrated function between tubules; Models allow perfusion/long term culture	Cells not included in the process; Tubule resolution as low as 20 µm but below 200 µm cell seeding is limited	[22,23,25]
Proximal tubule, glomerulus	Human primary kidney cells	dECM, gelatin, Hyaluronic acid, glycerol	Extrusion-based	Bioprinting of dECM-based bioinks	Developed bioinks suitable for primary cells; Reabsorption function observed on cells	dECM requires source organ; Limited functional readout demonstrated	[29]
Kidney tubulointerstitium	Primary murine epithelial cells, HUVECs	Alginate, pectin and gelatin	Extrusion-based (microfluidics core-shell)	Core-shell constructs with epithelial and endothelial concentrically bioprinted into shell and with a sacrificial gel core	Multiple configurations in core–shell format	Limited formation of cellular lumen	[14]
Kidney organoid model for drug testing	Induced pluripotent stem cell (iPSC)-derived kidney organoids	Biomaterial ink free	Extrusion bioprinting (piston-based)	High-throughput (96-well plate format) platform for organoid maturation and drug screening studies	Patterning with clear impact on differentiation and maturation of organoids	Bioprinting conformation limited in thickness; Absence of supporting biomaterials	[50]
Kidney organoid model for disease modelling, toxicity and improved differentiation	Embryonic or iPSC-derived kidney organoids	Biomaterial ink free	Liquid handling dispensing system	High-throughput organoid platform (96-well plate format)	Allows high throughput screenings for differentiation, toxicity and disease modeling	Dispensing approach without patterning control described	[75]

## Vasculature in kidney models

Renal vasculature is especially important in the kidney as the filtration of the blood from waste products and maintenance of homeostasis of the body fluids occurs in this organ [76]. Various renal *in vitro* models have been improved by the addition of a vascular tubule, in which both PT as vascular tubule were initially created by seeding an empty channel created with pluronic filaments as previously described [25]. In the model by Lin et al., the co-culture of PT epithelium and vascular endothelial cells allows for a crosstalk between compartments as concluded when exposed to hyperglycemic conditions (Figure 4E) [25]. Another approach for a tubular model is bioprinting of a fugitive ink with a monolayer or bilayer structure across the tubules length (Figure 5B,C) [20]. In the present study, the cells are included in the bioink formulations and dispensed with a triple coaxial nozzle where PT epithelial cells were printed around a shell layer of epithelial cells that on its turn were printed around pluronic as the core of the filament. These initial approaches demonstrate that it is possible to create vascular lumens with hundreds of microns in diameter and more research is needed to achieve dimensions of the kidney glomerulus and the microvessel network present throughout the tubulointerstitium, generally below 100 microns.

## Conclusion and future outlook

Over the past decade, progress has been made using 3D bioprinting methods to produce *in vitro* models of renal tissues and kidney-like constructs. Multiple bioprinting technologies have been investigated to allow an accurate deposition of biomaterials inks and bioinks to produce pre-designed constructs with micro and macro accuracy and resolution. Kidney models started to become more representative and showing some degree of function when primary cells or human cell lines are used. These models are frequently tested with nephrotoxic drugs showing to some extent the injury level for example in drug induced acute kidney injury.

Several challenges still persist to build fully functional *in vitro* models that can mimic mature organ, e.g., the choice of suitable cells and biomaterials, the bioprinting technique selection and manufacturing approach, and the need for post-manufacturing maturation in suitable physiological conditions. With the advances in developmental biology and more specifically the generation of renal organoids, it is observed that some initial studies where these rudiment structures are combined with bioprinting approaches to include vascularization and to ultimately achieve maturation and function of these structures [50,70,75]. The development of novel biomaterial inks that can be easily recognized and remodeled dynamically by cells is being exponentially investigated and these new alternatives will be essential to allow further progress of the bioprinted constructs [60]. Another essential point that researchers are addressing is the need for active perfusion of the printed constructs to ensure a sufficient exchange of nutrients and metabolic waste [70].

Future approaches will most likely explore combination of multiple bioprinting techniques in a single platform to allow a wider range of conditions possible from the single cell deposition to the macro-size multi-material and multi-cellular models. These models will be essential for general biological understanding of kidney development, maturation and function, to investigate renal diseases and as test-beds for drug development *in vitro*. These will also progressively help on addressing many challenges that lie ahead which remain unsolved allowing the step-by-step generation of new knowledge which will certainly contribute for future alternative therapies [6].

## Summary

- Bioprinting techniques have shown a great potential for the biofabrication of renal *in vitro* models but several challenges, such as the need for suitable biomaterial ink and cells for the formulation of bioinks need to be addressed.
- The post-printing maturation and the level of functionality of the constructs is still limited.
- Technological, biomaterial and biological developments will be further needed to achieve fully functional *in vitro* renal models.
- Bioprinted renal *in vitro* models will be essential to investigate drug nephrotoxicity, to mimic renal disease and to facilitate the development of new therapies.

## Abbreviations

AM: additive manufacturing
CKD: chronic kidney disease
dECM: decellularized ECM
DOD: droplet-on-demand
ECM: extracellular matrix
FDM: fused deposition modeling
GMEC: glomerulus microvascular endothelial cell
HUVEC: human umbilical vein endothelial cell
iPSCs: induced pluripotent stem cells
PCL: poly(ε-caprolactone)
PDMS: polydimethylsiloxane
PSC: pluripotent stem cell
PT: proximal tubule
PTECs: proximal tubule epithelial cells
